# 

**Published:** 1905-10

**Authors:** 


					﻿Sixty-first Year.
Vol. LXI.
OCTOBER, 1905.
No. 3
BUFFALO
MEDICALJOURNAL
JEstablisbed 1845 by AUSTIN FLINT, M. D.
’ '	EDITOR :
WILLIAM WARREN POTTER, 'M. D.
ASSISTANT EDITORS:
WILLIAM C. KRAUSS, M. D.
NELSON W. WILSON, M. D.
ASSOCIATE EDITORS:
JAMES WRIGHT PUTNAM, M. D.
JOHN PARMENTER, M. D.
HARVEY R. GAYLORD, M. D.
ERNEST WENDE, M. D.
JOHN A. MILLER, Ph. D.
MAUD J. FRYE, M. D.
				

